# Risk Factors for Band Keratopathy in Aphakic Eyes With Silicone Oil Tamponade for Open-Globe Injuries: A Multicenter Case-Control Study

**DOI:** 10.3389/fmed.2021.713599

**Published:** 2021-07-23

**Authors:** Kai He, Mengyu Liao, Yun Zhu, Bohao Cui, Haoyu Chen, Ting Wang, Nan Wu, Zhenggao Xie, Jing Luo, Yong Wei, Zhiliang Wang, Heding Zhou, Zhansheng Shen, Hua Yan

**Affiliations:** ^1^Department of Ophthalmology, Tianjin Medical University General Hospital, Tianjin Medical University, Tianjin, China; ^2^Department of Epidemiology and Biostatistics, School of Public Health, Tianjin Medical University, Tianjin, China; ^3^Department of Ophthalmology, Joint Shantou International Eye Center of Shantou University and the Chinese University of Hong Kong, Shantou, China; ^4^State Key Laboratory Cultivation Base, Shandong Provincial Key Laboratory of Ophthalmology, Department of Ophthalmology, Shandong Eye Institute, Eye Hospital of Shandong First Medical University, Shandong First Medical University & Shandong Academy of Medical Sciences, Jinan, China; ^5^Department of Ophthalmology, Southwest Hospital, Southwest Eye Hospital, Army Medical University, Chongqing, China; ^6^Department of Ophthalmology, Nanjing Drum Tower Hospital, The Affiliated Hospital of Nanjing University Medical School, Nanjing, China; ^7^Department of Ophthalmology, The Second Xiangya Hospital, Central South University, Changsha, China; ^8^The Eye Hospital of Wenzhou Medical University, Wenzhou, China; ^9^Department of Ophthalmology, Huashan Hospital of Fudan University, Shanghai, China; ^10^Department of Ophthalmology, Ningbo Eye Hospital, Wenzhou Medical University, Ningbo, China; ^11^Department of Ophthalmology, Zhengzhou Second Hospital, Zhengzhou, China

**Keywords:** band keratopathy, open-globe injuries, silicone oil tamponade, aphakic eye, zone of injury, silicone oil retention time

## Abstract

Band keratopathy (BK) is a common complication in aphakic eyes with silicone oil tamponade for open-globe injury (OGI), characterized by the grayish-white opacities in the cornea, resulting in a significantly decreased vision when extending to the visual axis. To identify the risk factors for BK in aphakic eyes following vitreoretinal surgical treatment with silicone oil tamponade for OGIs, we performed a multicenter case-control study. The incidence of BK was 28% (28/100 eyes). The multivariate binary logistic regression revealed the silicone oil retention time (SORT) ≥6 months and zone III injury were significant risk factors for BK. From the hierarchical interaction, SORT ≥6 months had a significant risk for BK in eyes with rupture, aniridia, and zone III injury, while zone III injury had a significant risk for BK in eyes with rupture, incomplete/complete iris, and SORT ≥6 months. By using restricted cubic splines with three knots at the 25th, 50th, and 75th centiles to model the association of SORT with BK, we also found a marked increase in the risk for BK at ≥10 months and a slow increase after 6 months, but almost stable within 4–6 months.

## Introduction

Open globe injury (OGI) is a common cause of unilateral visual impairment and blindness worldwide (1, 2). Severe and complicated ocular trauma can break the iris-lens barrier and affect both the anterior and posterior segments, leading to an enormous variety of anterior segment injuries of the eye, including open corneal trauma, complete or incomplete iris defect, traumatic cataract, ciliary body detachment, and cyclodialysis (3, 4). With the advancement of surgical techniques, vitrectomy combined with anterior segment surgical procedures has become an effective method in treating severe and complicated cases. Retinal detachment (RD) is a vision-threatening complication of OGI (5). For patients with complicated RD in OGI, silicone oil tamponade is necessary, even in the long term in some patients. However, it is well-known that silicone oil tamponade has some complications such as band keratopathy (BK), which is characterized by the deposition of grayish-white opacities in the superficial layers of the cornea, resulting in a significantly decreased vision when the opacities extend to the visual axis. Patients with BK may also suffer from eye irritation and pain (6).

Morphis et al. (7) reported that BK (8%) and corneal decompensation (12%) occurred in eyes with silicone oil tamponade for more than 12 months. Goezinne et al. (8) also concluded that intact natural or artificial lens-iris diaphragms protect corneal endothelial cells from damage by long-term silicone oil tamponade. The lack of the natural barrier, BK, and corneal endothelial decompensation occur more frequently in eyes with traumatic aphakia and aniridia. Secondary intraocular pressure (IOP) changes and silicone oil entering the anterior chamber should also be considered. Shah et al. (9) found that the incidences of postoperative corneal decompensation, BK, ocular hypertension, and hypotony were 4.7, 6.4, 9.4, and 21.9%, respectively, after silicone oil removal. Yüksel et al. (10) placed silicone oil barrier sutures in aphakic eyes with iris defects; hypotony and BK still occurred in 31% of eyes. However, few published reports have detailed the risks of BK in traumatic eyes after vitreoretinal surgical treatment combined with silicone oil tamponade for OGIs with aphakia.

In this study, we analyzed the risks of BK in traumatic eyes following vitreoretinal surgical treatment with silicone oil tamponade for OGIs with aphakia using a multicenter case-control study.

## Methods

### Patients

**Figure 1:**
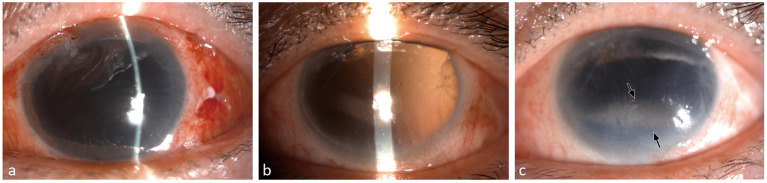
Band keratopathy in a patient. This was a 58-year-old female patient who got eye injured in zone III and diagnosed as rupture with aphakia and aniridia. After the secondary vitrectomy management, the visual acuity was light perception and the intraocular pressure was 10 mmHg. The postoperative corneal transparent was observed at 1 month **(a)** and 3 months **(b)**. Band keratopathy occurred after silicone oil tamponade for 8 months (**c**, black arrow).

The medical records of all patients with OGI undergoing vitreoretinal surgical treatment with silicone oil tamponade at the Department of Ophthalmology of 10 hospitals from all over China between 2014 and 2020 were screened. The inclusion criteria were OGI eyes with aphakia or posterior capsule rupture treated with pars plana vitrectomy combined with silicone oil tamponade. On the other hand, the exclusion criteria included a history of previous intraocular surgery, keratopathy, postoperative endophthalmitis, incomplete clinical data, and follow-up time of <3 months. Under slit-lamp microscopy, eyes with BK were observed with obvious band-shaped grayish to whitish opacities in the superficial layers of the cornea, most frequently in the interpalpebral zone (Figure 1). The patients were divided into two groups according to the outcome of BK: BK and non-BK groups. This study was conducted in accordance with the tenets of the Declaration of Helsinki and was approved by the Tianjin Medical University General Hospital Ethics Committee. The requirement for informed consent was waived because of the retrospective nature of the study.

### Data Collection

This was a multicenter, case-control study. The demographic information of the patients such as sex and age, and clinical characteristics, including diagnosis, zone of injury, iris status, best-corrected visual acuity (BCVA), IOP, and silicone oil retention time (SORT), were collected from medical records. The BCVAs were measured using Snellen charts with standardized procedures by certified masked research officers, and IOP was measured using non-contact IOP measurements (CT80, Topcon, Japan). According to the Birmingham Eye Trauma Terminology (4), the zone of injury was coded as an ordinal variable: Zone I, cornea only; Zone II, 5 mm posterior to the limbus; and Zone III, ≥5 mm posterior to the limbus. All ophthalmic evaluations were reassessed by senior ophthalmologists before, during, and after the surgery. All the surgeons involved were experienced and performed a standard three-port vitrectomy combined with silicone oil tamponade.

### Statistical Analysis

The analysis was performed using Statistical Product and Service Solutions (SPSS) version 25.0 for Windows (IBM Corp, Armonk, NY, USA). Univariate analysis using the chi-square test or Fisher's exact test was performed to determine the significance of categorical variables. An independent *t*-test was used to compare continuous variables. Variables that were significant in the univariate analysis or clinically meaningful indicators were examined using binary logistic regression analysis to predict independent factors. Hierarchical interaction analysis was used to determine the association between BK and risk factors. All statistical tests were two-tailed, and statistical significance was defined as *P* < 0.05.

For more detailed analyses of the time-response trends, restricted cubic splines (RCS) with three knots at the 25th, 50th, and 75th centiles were used to flexibly model the association of silicone oil retention time with the outcome of BK; the model was adjusted for IOP, sex, diagnosis, zone of injury, and iris status). This was conducted using SAS version 9.4 (SAS Institute, Inc., Cary, NC, USA) (11).

## Results

A total of 100 patients (100 eyes), who met all the inclusion criteria and did not meet the exclusion criteria, were enrolled in the study. During the follow-up period, 28 eyes (28%) developed BK. The demographic and clinical characteristics of the study population are shown in Table 1. There were no significant differences in sex and age between the BK and non-BK groups. The SORT was significantly longer in the BK group (13.96 ± 10.71 months) than in the non-BK group (7.86 ± 6.81 months, *P* = 0.001). In the BK group, zone III injured eyes accounted for 75.0%, which was higher than that in the non-BK group (38.9%, *P* = 0.01). Additionally, the BK eyes were more likely to be with rupture (85.7%) and aniridia (42.9%), although these results were not statistically significant.

**Table 1:** Demographic and clinical characteristics of the study population.

**Characteristics**	**Non-BK (*n =* 72)**	**BK (*n =* 28)**	***P-*value**
Sex (*n*, %)			0.46
Male	61 (84.7%)	22 (78.6%)	
Female	11 (15.3%)	6 (21.4%)	
Age (year)	44.78 ± 14.69	48.29 ± 15.78	0.29
Diagnosis (*n*, %)			
Rupture	43 (59.7%)	24 (85.7%)	0.92
Laceration	29 (40.3%)	4 (14.3%)	
Zone of injury (*n*, %)			
Zone I/II	44 (61.1%)	7 (25.0%)	0.01^*^
Zone III	28 (38.9%)	21 (75.0%)	
Iris status (*n*, %)			
Aniridia	16 (22.2%)	12 (42.9%)	0.11
Partial iris	28 (38.9%)	9 (32.1%)	
Complete iris	28 (38.9%)	7 (25%)	
Best-corrected visual acuity (*n*, %)			
No light perception	8 (28.6%)	1 (3.6%)	0.33
Light perception	52 (72.2%)	24 (85.7%)	
Hand motion or better	12 (16.7%)	3 (10.7%)	
Silicone oil retention time (month)	7.86 ± 6.81	13.96± 10.71	0.001^*^
Intraocular pressure (mmHg)	13.53 ± 6.99	12.12 ± 4.62	0.33

*Values were presented as mean ± standard deviation or number (percentage)*.

* *P < 0.05. BK, band keratopathy*.

Table 2 provides the adjusted odd ratios (ORs) and 95% confidence intervals (CIs) for BK using a multivariable logistic regression analysis. After adjusting for age and other selected variables, the results showed that age (OR = 1.04; 95% CI: 1.00–1.08, *P* = 0.08) was close to a borderline, and SORT (OR = 1.32; 95% CI: 1.06–1.21, *P* = 0.001) and zone of injury (OR = 6.88; 95% CI: 1.94–24.44, *P* = 0.001) remained as independent risk factors for BK.

**Table 2:** Odds ratios for the association between risk factors and band keratopathy.

**Characteristics**	**BK/Non-BK**	**OR**	**95%CI**	***P-*value**
Age (year)	48.29 ± 15.78/44.78 ± 14.69	1.04	1.00–1.08	0.08
Zone of injury (*n*)				
Zone I/II	7/44	1.00		
Zone III	21/28	6.88	1.94–24.44	0.001^*^
Diagnosis (*n*)				
Rupture	24/43	1.00		
Laceration	4/29	0.35	0.08–1.62	0.18
Iris status (*n*)				
Aniridia	12/16	1.00		
Incomplete iris	9/28	0.50	0.14–1.78	0.28
Complete iris	7/28	1.04	0.25–4.32	0.96
Silicone oil retention time (month)	13.96 ± 10.71/7.86 ± 6.81	1.32	1.06–1.21	0.001^*^
Intraocular pressure (mmHg)	12.12 ± 4.62/13.53 ± 6.99	0.93	0.85–1.03	0.17

*Variables were mutually adjusted for each other in the multivariable logistic regression models*.

* *P < 0.05. BK, band keratopathy; OR, odds ratio; CI, confidence intervals*.

The binary logistic regression model was repeated for BK between strata defined by SORT (Table 3) and the zone of injury (Table 4). Specifically, the risk of BK associated with silicone oil retention over 6 months was limited to the eyes diagnosed with rupture (OR = 5.08; 95% CI: 1.42–18.16, *P* = 0.01), aniridia (OR = 9.84; 95% CI: 1.26–76.74, *P* = 0.01), and zone III injury (OR = 7.74; 95% CI: 1.76–34.10, *P* = 0.03). Moreover, the impact of zone III injury was more marked in the eyes diagnosed with rupture (OR = 6.84; 95% CI: 1.69–27.69 *P* = 0.01), incomplete/complete iris (OR = 13.80; 95% CI: 2.76–69.08, *P* = 0.001), and silicone oil retention over 6 months (OR = 7.71; 95% CI: 1.73–34.73, *P* = 0.01).

**Table 3:** Association between the time of silicone oil retention and band keratopathy stratified by diagnosis, injury zone, and iris status.

	**BK/Non-BK (*n*)**	**OR (95% CI)**			***P*_**interaction**_**
		**3–6 months**	**>6 months**	***P-*value**	
Diagnosis					
Rupture	24/43	1.00	5.08 (1.42–18.16)	0.01^*^	
Laceration	4/29	1.00	5.06 (0.33–77.54)	0.25	0.92
Zone of injury					
Zone I/II	7/48	1.00	4.15 (0.41–41.80)	0.23	
Zone III	21/28	1.00	7.74 (1.76–34.10)	0.01^*^	0.40
Iris status					
Aniridia	12/16	1.00	9.84 (1.26–76.74)	0.03^*^	
Incomplete/complete iris	16/56	1.00	3.01 (0.76–11.97)	0.12	0.20

* *P < 0.05. BK, band keratopathy; OR, odds ratio; CI, confidence intervals*.

**Table 4:** Association between the zone of injury and band keratopathy stratified by diagnosis, time of silicone oil retention, and iris status.

	**BK/Non-BK (n)**	**OR (95% CI)**			***P*_**interaction**_**
		**Zone I/II**	**Zone III**	***P-*value**	
Diagnosis					
Rupture	24/43	1.00	6.84 (1.69–27.69)	0.01^*^	
Laceration	4/29	1.00	2.55 (0.15–44.89)	0.52	0.76
Time of silicone oil retention					
3–6 months	9/31	1.00	3.70 (0.53–26.10)	0.19	
>6 months	19/30	1.00	7.71 (1.73–34.73)	0.01^*^	0.40
Iris status					
Aniridia	12/16	1.00	2.06 (0.26–16.55)	0.50	
Incomplete/complete iris	16/56	1.00	13.80 (2.76–69.08)	0.001^*^	0.40

* *P < 0.05. BK, band keratopathy; OR, odds ratio; CI, confidence intervals*.

**Figure 2:**
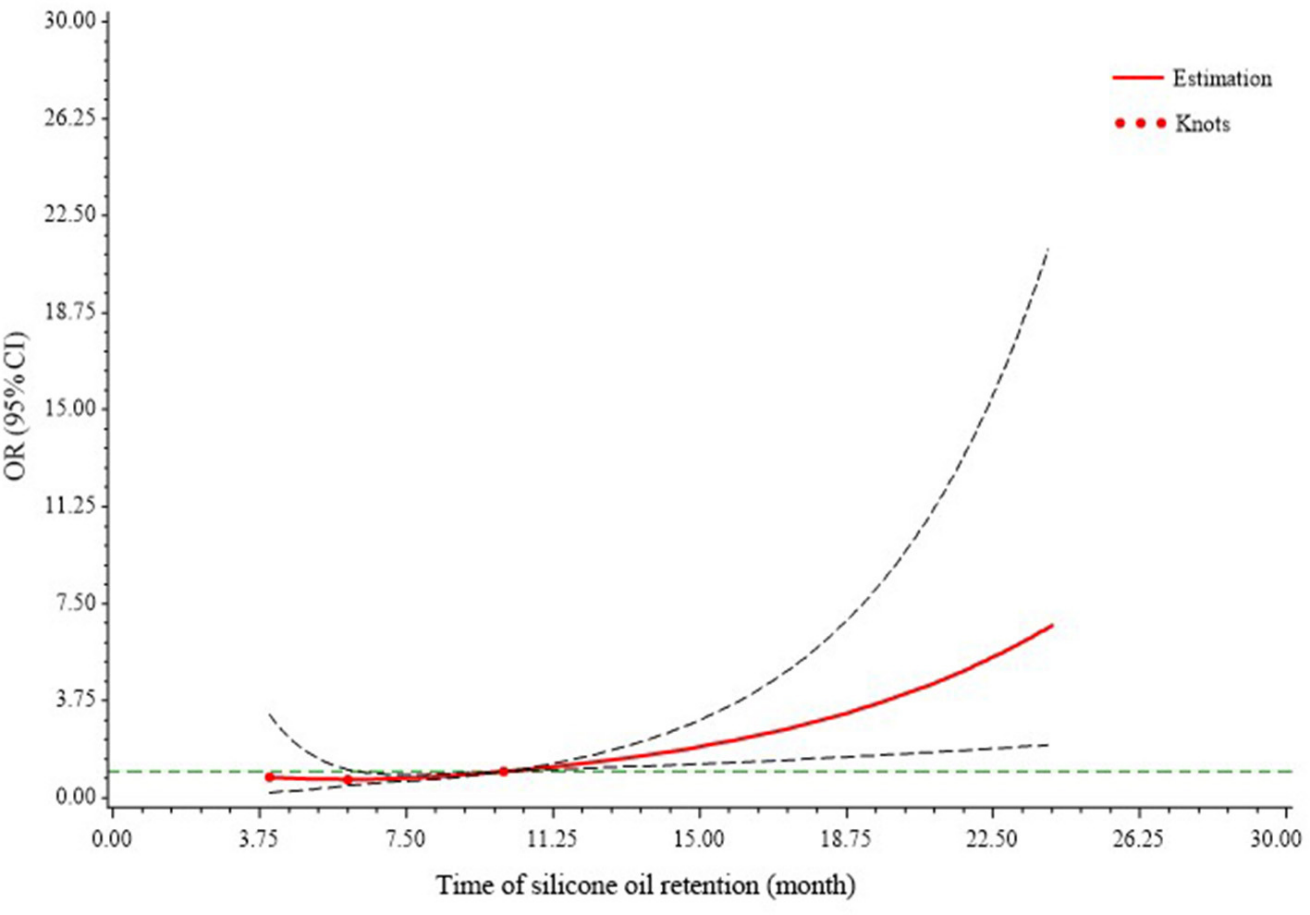
Association between band keratopathy and silicone oil retention time. Restricted cubic splines with three knots at the 25th, 50th, and 75th centiles were used to model the association between band keratopathy and silicone oil retention time. This model was adjusted for intraocular pressure, sex, diagnosis, zone of injury, and iris status. The 95% confidence intervals are indicated by the black dashed lines. The risk of band keratopathy increases with time and remains almost stable within 6 months, increases slowly at 6–10 months, and increases sharply after 10 months postoperatively. CI, confidence interval; OR, odds ratio.

To evaluate the risk of BK with silicone oil retention time, we developed an RCS logistic regression model to fit all the data, which was adjusted for IOP, sex, diagnosis, zone of injury, and iris status (Figure 2). The 25th, 50th, and 75th centiles were 4, 6, and 10 months, respectively. There was an approximately linear correlation between SORT and the risk of BK, and a marked increase in risk for BK was observed ≥10 months, but minimal elevation in risk within 6 months. Within 6–10 months postoperatively, the risk of BK increases slowly with time.

## Discussion

The present study focused on BK in the eyes following vitreoretinal surgical treatment with silicone oil tamponade for OGI with aphakia and aimed to identify the risk factors for BK. We collected and analyzed the clinical data of 100 eyes from 10 hospitals across China. We found that silicone oil retention time over 6 months and zone III of OGI were independent risk factors for BK, with the risk of BK increasing sharply with time after 10 months postoperatively.

BK is a common corneal degenerative disease characterized by calcium deposition in the superficial layers of the cornea, including the epithelial basement membrane, basal epithelium, and Bowman's membrane (6). Although the pathophysiology of BK is multifactorial, many studies have reported that silicone oil plays an important role in the development of BK (7, 12, 13). With the advancement of vitreoretinal surgical techniques, silicone oil has been widely used as a vitreous substitute in various diseases and situations because of its chemical stability and special physical properties (14–17), especially in complex trauma cases (18). The present study found that silicone oil tamponade over 6 months was an independent risk factor for BK. Silicone oil is usually removed 3–6 months postoperatively. However, severe traumatic eyes with extensive retinal detachment may require sustained or refilled silicone oil to reattach the retina. Along with long-term silicone tamponade, the chance for oil to contact the corneal endothelium is increasing. Studies have shown that it can be related to the tissue toxic reaction of some components in silicone oil, and another hypothesis is that pH changes are caused by decreased flow across the corneal tissue (19). Berker et al. (20) observed anterior dislocation of silicone oil (9.5%) in patients with proliferative vitreoretinopathy. Szaflik et al. (21) also described confocal microscopy observations of the eyes with silicone oil in the anterior chamber and revealed alterations in the upper part of the cornea, which is the part most exposed to the silicone oil, with “multidot” lesions and lesions with thickening cell borders. Furthermore, Dooley et al. (22) reported that corneal pathology (13.8%) and anterior segment emulsification (8.3%) occurred in eyes with long-term heavy silicone oil tamponade. In contrast, incidences of postoperative corneal decompensation (4.7%) and band-shaped keratopathy (6.4%) were lower after silicone oil was removed (9).

In this study, the other independent risk factor for BK was zone III of the OGI, which suggests that the globe opening location would extend more posteriorly than the pars plana (4). OGIs with zone III injury are particularly serious because they are more likely to be associated with RD and worse final visual acuity (23, 24). Knyazer et al. (25) described that eyelid injury, cornea lamellar lacerations or abrasions, iris deformity, and many other signs were associated with a poor prognosis of the eyes with zone III injury. Here, we found a higher rate of BK (21/49 eyes, 48.9%) in OGI with zone III injury. A possible explanation is that zone III of the OGI aggravated the inflammatory response in the anterior segment, leading to sustained damage to the corneal tissues. In uveitis, inadequate control of eye inflammation can result in permanent and severe ocular complications, in which BK is common (26). Similarly, in severe OGIs, excessive, or persistent inflammation may exacerbate corneal damage. Additionally, structural damage to the ciliary body decreases the generation of the aqueous humor; thus, insufficient oxygen and other important nutrients were provided to the cornea, which affects the progression of corneal repair and recovery. Better ciliary body function might promote aqueous humor secretion and nourish the cornea while reducing the occurrence of BK.

From the results of the hierarchical interaction analysis in our study, silicone oil retention over 6 months had a significant risk for BK in eyes with aniridia, while zone III injury had a significant risk for BK in eyes with incomplete/complete iris. Iris status seemed to play a different role in the risk of BK. Evidently, the chance for silicone oil to enter the anterior chamber to contact the corneal endothelium would greatly increase without the barrier of the iris. To prevent BK, Gentile and Eliott (27) first introduced the technique “Silicone Oil Retention Sutures,” an effective means to prevent oil-corneal touch in eyes with aphakic and aniridia. In contrast, the remaining iris (either incomplete or complete) possibly play the role of main source of inflammatory mediators. Aketa et al. (28) also reported that the levels of aqueous protein and cytokines in eyes with iris damage were significantly higher than in eyes without iris damage.

The major innovation of this study was the creative application of the RCS model in showing the increasing risk of BK with silicone oil retention time. These results have instructive clinical significance and suggest that 10 months is a key time-point. For patients with severe ocular trauma following vitrectomy combined with silicone oil tamponade, a thorough examination and appropriate intervention is needed for BK during follow-up after 10 months postoperatively.

There were some limitations to this study. We focused on the risk factors for BK after vitrectomy and silicone oil tamponade. However, the effects of ocular trauma on the cornea were not compared and analyzed, which may cause a bias in the risk of BK in OGIs. Moreover, we did not analyze the location, shape, and occurrence time of BK due to the lack of sufficient details from each department. The high variability of the presentations of ocular trauma injuries was a limitation of the study, while the large number of patients and the standard surgical procedure made the conclusions more robust.

In summary, the results of our study are reliable and provide important insights into the risk factors for BK in eyes following vitreoretinal surgical treatment with silicone oil tamponade for OGIs with aphakia because of the limited number of previously published reports in this field. The risks of BK and simulated SORT can also be used for clinical guidance.

## Data Availability Statement

The raw data supporting the conclusions of this article will be made available by the authors, without undue reservation.

## Ethics Statement

The studies involving human participants were reviewed and approved by Tianjin Medical University General Hospital. Written informed consent to participate in this study was provided by the participants' legal guardian/next of kin. Written informed consent was obtained from the individual(s) for the publication of any potentially identifiable images or data included in this article.

## Author Contributions

KH, ML, and HY designed this study. KH, ML, BC, HC, TW, NW, ZX, JL, YW, ZW, HZ, and ZS collected and measured data. KH, YZ, and HY analyzed and interpreted the data set. KH and ML wrote this article. HC and HY revised the manuscript. All authors reviewed and approved the final manuscript.

## Conflict of Interest

The authors declare that the research was conducted in the absence of any commercial or financial relationships that could be construed as a potential conflict of interest.

## Publisher's Note

All claims expressed in this article are solely those of the authors and do not necessarily represent those of their affiliated organizations, or those of the publisher, the editors and the reviewers. Any product that may be evaluated in this article, or claim that may be made by its manufacturer, is not guaranteed or endorsed by the publisher.
